# The antidepressant-like effects of paeoniflorin in mouse models

**DOI:** 10.3892/etm.2013.925

**Published:** 2013-01-25

**Authors:** FENGMEI QIU, XIAOMING ZHONG, QINGQIU MAO, ZHEN HUANG

**Affiliations:** 1 College of Pharmacy, Zhejiang Chinese Medicine University, Hangzhou, Zhejiang 310053;; 2 School of Chinese Medicine, The Chinese University of Hong Kong, Shatin, Hong Kong 999077, SAR, P.R. China

**Keywords:** paeoniflorin, forced swimming, tail suspension, locomotor activity, reserpine, antidepressant-like effect, neurotransmitter

## Abstract

Peony is often used in Chinese herbal medicine for the treatment of depression-like disorders. Our previous studies have demonstrated that the total glycosides of peony exert antidepressant-like effects in animal models. Paeoniflorin is the main active glycoside of peony. The aim of this study was to evaluate the antidepressant-like effects of paeoniflorin in mice, as well as its active mechanisms. The results revealed that intraperitoneally injected paeoniflorin significantly reduced the duration of immobility in forced swimming and tail suspension tests. The doses that affected the immobility response did not affect locomotor activity. Furthermore, paeoniflorin antagonized reserpine-induced ptosis, akinesia and hypothermia. Paeoniflorin also significantly increased the levels of serotonin (5-HT) and its metabolite 5-hydroxyindoleacetic acid (5-HIAA) in the hippocampus. These results suggest that the upregulation of serotonergic systems may be an important mechanism for the antidepressant-like effects of paeoniflorin in mice.

## Introduction

Depression is a commonly occurring, debilitating and life-threatening psychiatric disorder. Various antidepressants, including tricyclic antidepressants, monoamine oxidase inhibitors and noradrenaline (NA) reuptake inhibitors, are widely available in the pharmaceutical market. However, the majority of these antidepressants have undesirable side-effects (1-3). Consequently, new antidepressants are sought. The root of *Paeonia lactiflora* Pall. (*Ranunculaceae*), commonly known as peony, is a commonly used in herbal medicines in China, Korea and Japan. It is a component herb of numerous traditional formulae, including Jiaweisinisan and Dang Gui Shao Yao San, prescribed for the treatment of depression-like disorders (4,5). The antidepressant-like effect of the total glycoside fraction of peony has also been observed in mice exposed to chronic unpredictable stress (6). Paeoniflorin, the main and active component of peony, has been widely studied as an antioxidant, anticonvulsant, antithrombotic agent, cognition enhancer or learning impairment-attenuating agent and neuroprotecting agent (7-13). However, there is no information available regarding the antidepressant activity of paeoni-florin following intraperitoneal injection in mice (14). In the present study, we assessed the antidepressant-like effects of paeoniflorin and its mechanisms by means of behavioural and pharmacological procedures.

## Materials and methods

### Animals

Male Institute of Cancer Research (ICR) mice (18–22 g) were obtained from the Laboratory Animal Unit of Zhejiang Chinese Medicine University (Zhejiang, China). The animals were housed five per cage and acclimatized to a colony room with controlled ambient temperature (24±1°C), humidity (50±10%) and a 12 h light/dark cycle. They were fed a standard diet and water *ad libitum* and were left to acclimate for 4 days before use in experiments. The use of experimental mice was approved by the Animal Experimentation Ethics Committee of the Zhejiang Chinese Medicine University and conducted in accordance with the National Institutes of Health (NIH) Principles of Laboratory Animal Care (publication No. 80-23, revised 1996).

### Drugs

Paeoniflorin (purity >98%) was purchased from Guizhou Dida Technology Co., Ltd. (Zhejiang, China). Imipramine hydrochloride was purchased from Sigma-Aldrich (St. Louis, MO, USA) as a positive control. Reserpine was purchased from Bangmin (Guangzhou, China) and 5-hydroxytryptamine (5-HT), NA, dopamine (DA) and 5-hydroxyindoleacetic acid (5-HIAA) were purchased from Sigma-Aldrich. For intraperitoneal injection, paeoniflorin, imipramine and reserpine were separately dissolved in 0.9% normal saline and diluted to the desired concentration on the day of testing. In this study, various doses of paeoniflorin (10, 20 and 40 mg/kg) and imipramine (10 mg/kg) were injected intraperitoneally (i.p.) 60 min before testing.

### Tail suspension test

The tail suspension test was based on the method of Steru *et al* (15). Animals were suspended 50 cm above the floor by means of an adhesive tape, placed ∼1 cm from the tip of the tail. The duration of immobility was recorded during the last 4 min of the 6-min testing period. Mice were considered immobile only when they hung passively and completely motionless.

### Forced swimming test

The forced swimming test was similar to those described previously (16,17). The mice were individually placed into glass cylinders (height, 25 cm; diameter, 10 cm) containing 10 cm of water (22±1°C). The duration of immobility was defined as the time the mouse spent without struggling, floating motionless or making only small movements necessary to keep its head above water for the 6-min period. The water was exchanged following each trial.

### Locomotor activity

Locomotor activity was studied using an open-field test which was performed on mice using a slightly modified method (18,19). Briefly, the locomotor activity of the mice was measured using a box (30×30×15 cm) with the floor divided into 25 squares illuminated with light from the ceiling. Mice were placed in the central square and the total number of squares entered was recorded for 2 min. The open field arena was cleaned following each trial.

### Tests of reserpine-induced ptosis, akinesia and hypothermia

The tests of reserpine-induced ptosis, akinesia and hypothermia were in accordance with those of Bourin *et al* (20). The mice were treated with reserpine (2.5 mg/kg, i.p.) 10 min after the administration of paeoniflorin. Three parameters of akinesia, the degree of palpebral ptosis and the rectal temperature were recorded at 1 h, 1 h and 4 h, respectively, after the administration of reserpine. The degree of palpebral ptosis was evaluated according to the following rating scale: 0, eyes open; 1, eyes one-quarter closed; 2, eyes half closed; 3, eyes three-quarters closed and 4, eyes completely closed. To measure akinesia, mice were placed in the center of a circle (diameter, 7.5 cm). The total time the mice remained within the circle during a 1-min period was counted.

### Monoamine neurotransmitter assay

Levels of 5-HT, NA, DA and 5-HIAA in the hippocampus were measured by high-performance liquid chromatography (HPLC) coupled with an electrochemical detector as described previously (4). Briefly, each frozen tissue sample was homogenized in 0.4 M perchloric acid (solution A). The homogenate was stored on ice for 1 h and then centrifuged at 12,000 × g (4°C) for 20 min. The pellet was discarded. A 160-*μ*l aliquot of the supernatant was added to 80 *μ*l solution B [(containing 0.2 M potassium citrate, 0.3 M dipotassium hydrogen phosphate and 0.2 M ethylenediamine tetraacetic acid (EDTA)]. The mixture was stored on ice for 1 h and then centrifuged at 12,000 × g (4°C) for 20 min. Then, 20 *μ*l of the resultant supernatant was directly injected into an ESA liquid chromatography system equipped with a reversed-phase C18 column (150×4.6 mm, 5 *μ*m) and an electrochemical detector (ESA CoulArray, Chelmsford, MA, USA). The detector was set at 450 mV. The mobile phase consisted of 125 mM citric acid-sodium citrate (pH 4.3), 0.1 mM EDTA, 1.2 mM sodium octanesulfonate and 16% methanol. The flow rate was 1.0 ml/min. NA, 5-HT, DA and 5-HIAA were identified and quantified by comparing their retention times and peak areas to those of standard solutions. The contents of 5-HT, NA, DA and 5-HIAA were expressed as ng/g in wet weight tissue.

### Statistical analysis

The results were expressed as the mean ± standard error of the mean. All data were analyzed statistically using one-way analysis of variance, followed by Dunnett’s test. P<0.05 was considered to indicate a statistically significant difference.

## Results

### Paeoniflorin decreases the duration of immobility in the tail suspension test

**Figure 1:**
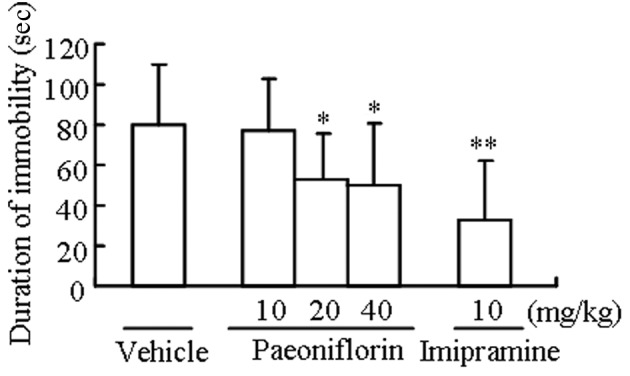
Effect of paeoniflorin on the duration of immobility in the tail suspension test. Values are expressed as the mean ± standard error of the mean (SEM) with 11 mice in each group. Data analysis was performed using Dunnett’s test. ^*^P<0.05 and ^**^P<0.01 vs. the vehicle control group.

Paeniflorin at doses of 20 and 40 mg/kg significantly decreased the duration of immobility in the tail suspension test compared with the control group (P<0.05). Imipramine (10 mg/kg) treatment also had a significant effect on immobility (P<0.01; Fig. 1).

### Paeoniflorin decreases the duration of immobility in the forced swimming test

**Figure 2:**
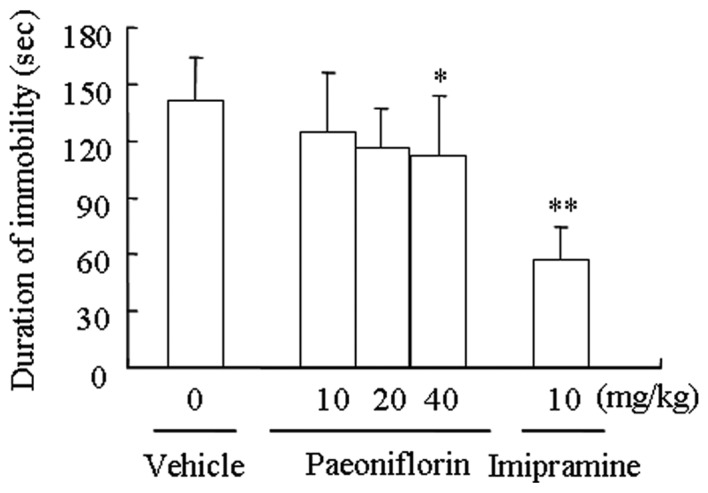
Effects of paeoniflorin on the duration of immobility in the forced swimming test. Values are expressed as the mean ± standard error of the mean (SEM) with 10 mice in each group. Data analysis was performed using Dunnett’s test. ^*^P<0.05 and ^**^P<0.01 vs. the vehicle control group.

Paeoniflorin (40 mg/kg) significantly inhibited immobility in the forced swimming test (P<0.05). Imipramine (10 mg/kg) treatment also decreased the duration of immobility (P<0.01; Fig. 2).

### Paeoniflorin has no effect on locomotor activity

**Figure 3:**
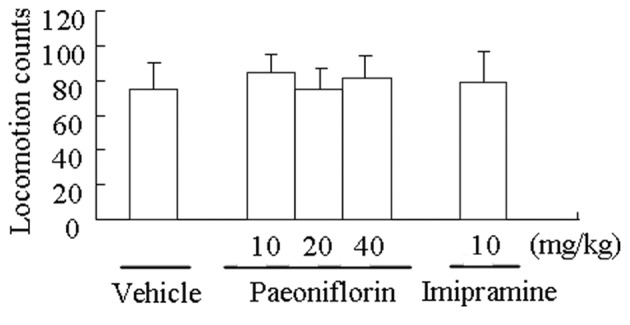
Effects of paeoniflorin on locomotor activity in mice. Values are expressed as the mean ± standard error of the mean (SEM) with 10 mice in each group. Data analysis was performed using Dunnett’s test. There were no significant differences when compared with the vehicle control group.

The effect of paeoniflorin on the locomotor activity of mice is shown in Fig. 3. Neither paeoniflorin nor imipramine affected locomotor activity at the doses that significantly reduced the immobility response in the tail suspension and forced swimming tests.

### Paeoniflorin attenuates reserpine-induced ptosis, akinesia and hypothermia

The effects of paeoniflorin on reserpine-induced ptosis, akinesia and hypothermia are shown in Table I. The paeoniflorin treatment at doses of 20 and 40 mg/kg significantly decreased the scores of reserpine-induced ptosis (P<0.01). The paeoniflorin treatment at 40 mg/kg significantly reduced the time of reserpine-induced akinesia (P<0.05) and significantly antagonized reserpine-induced hypothermia compared with vehicle treatment (P<0.05). Imipramine at a dose of 10 mg/kg significantly antagonized the hypothermia, ptosis and akinesia induced by reserpine (P<0.05, P<0.01 and P<0.05, respectively).

### Paeoniflorin increases the concentrations of monoamine neurotransmitters and a major metabolite in the hippocampus

**Figure 4:**
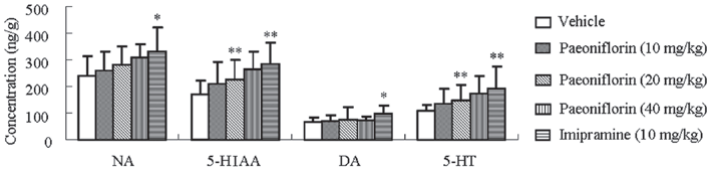
Effects of paeoniflorin on monoamine neurotransmitters and a major metabolite of 5-HT in the hippocampus. Values are the mean ± standard error of the mean (SEM) with 8 mice in each group. Data analysis was performed using Dunnett’s test. ^*^P<0.05 and ^**^P<0.01 vs. the vehicle control group. NA, noradrenaline; 5-HIAA, 5-hydroxyindoleacetic acid; DA, dopamine; 5-HT, 5-hydroxytryptamine.

The effect of paeoniflorin on hippocampal monoamine neurotransmitters and a major metabolite of 5-HT in mice is presented in Fig. 4. Paeoniflorin (40 mg/kg) treatment significantly increased the concentration of 5-HIAA and 5-HT in the hippocampus (P<0.01) compared with vehicle treatment, while imipramine treatment significantly affected hippocampal NA, 5-HIAA, DA and 5-HT concentrations (P<0.05, P<0.01, P<0.05 and P<0.01, respectively).

## Discussion

The tail suspension and forced swimming tests are widely used for the screening of antidepressant activity (16,18). The forced swimming and tail suspension-induced state of immobility in animals is similar to human depression and is amenable to reversal by antidepressant drugs (16,18). These animal models are based on the despair or helpless behaviour to an inescapable and confined space in animals. The present results demonstrated that paeoniflorin induces significant antidepressant-like effects in these models. The decrease in immobility time was dose-dependent in two models.

In these behavioural tests, false-positive results are occasionally obtained for agents that stimulate locomotor activity (21). Therefore, we determined whether paeoniflorin has excitatory or inhibitory actions on the central nervous system. In our study, paeoniflorin had no effect on the spontaneous locomotor activity of mice, indicating that paeoniflorin had no excitatory or inhibitory action on the central nervous system in effective doses, which eliminates the probability of false-positive results in the tail suspension and forced swimming tests. This finding suggests that the reduction of immobility time elicited by paeoniflorin treatment in the tail suspension and forced swimming tests is likely to be due to a psychomotor-stimulant effect and not a psychomotor-stimulant action.

Reserpine is an antihypertensive drug that depletes neuronal storage granules of biogenic amines in the brains of rodents and produces a clinically significant depression-like state (22). Mice become hypothermic, akinetic and diarrhetic, with eyelid drooping, in response to reserpine. Reserpine irreversibly inhibits the vesicular uptake of monoamine neurotransmitters, including NA, DA and 5-HT (21,23). The symptoms are reversed by major classes of antidepressant drugs. The results obtained in the present study demonstrate that paeoniflorin dose-dependently antagonizes the ptosis, akinesia and hypothermia induced by reserpine in mice, which indicates that paeoniflorin has an antidepressant-like effect and may have an effect on monoamine neurotransmitters. We then explored the antidepressant mechanism of paeoniflorin by determining the levels of monomine neurotransmitters in the hippocampus by HPLC-electrochemical detection (ECD). The results revealed that paeoniflorin increased the levels of monoamine neurotransmitters in the mouse hippocampus. This observation suggests that the antidepressant-like effects of paeoniflorin may be caused by the preservation of monoamine neurotransmitters.

In a conclusion, upregulation of serotonergic systems may be an important mechanism in the antidepressant-like effects of paeoniflorin in mice.

## Figures and Tables

**Table I:** Antagonism of paeoniflorin on the reserpine-induced ptosis, akinesia and hypothermia in mice.

Treatment	Rectal temperature (°C)	Scores of ptosis	Akinesia (sec)
Vehicle	32.00±0.0	3.5±0.7	60.0±0
Paeoniflorin (10 mg/kg)	32.05±0.2	3.5±0.5	54.8±17
Paeoniflorin (20 mg/kg)	32.03±0.1	2.1±1.3^b^	43.0±27
Paeoniflorin (40 mg/kg)	32.07±0.1^a^	2.0±1.3^b^	32.4±29^a^
Imipramine (10 mg/kg)	34.94±0.6^a^	0.7±0.8^b^	32.3±23^a^

Values are the mean ± standard error of the mean (SEM) with 10 mice in each group. Data analysis was performed using Dunnett’s test.

a P<0.05 and

b P<0.01 vs. the vehicle control group.
